# Correction: Reliability of an automatic classifier for brain enlarged perivascular spaces burden and comparison with human performance

**DOI:** 10.1042/CS20170051_COR

**Published:** 2017-12-14

**Authors:** Víctor González-Castro, María del C. Valdés Hernández, Francesca M. Chappell, Paul A. Armitage, Stephen Makin, Joanna M. Wardlaw

***Clin. Sci.*, 2017, volume 131, issue 13, pages 1465–1481, https://doi.org/10.1042/CS20170051**

In addition to the funding acknowledged in the published article, M.C.V.H. and J.M.W. also received funds for this work from the European Union Horizon 2020 [grant number PHC-03-15, project number: 666881, ‘SVDs@Target’], Innovate UK (Ref.46917-348,146); and the Fondation Leducq Network for the Study of Perivascular Spaces in Small Vessel Disease [reference number: 16 CVD 05]. These three funding sources are also gratefully acknowledged.

